# Clinical Outcomes of the Allegra Transcatheter System in Bioprosthetic Aortic Valve Failure: A Multicentre Valve‐in‐Valve Registry

**DOI:** 10.1002/ccd.70811

**Published:** 2026-08-21

**Authors:** Hilal Khan, Mohamed Ali, Abdalazeem Ibrahem, Karim Ratib, Nur Ilia Mohd Zaki, Freidoon Keshavarzi, Alok Shah, Hesham Abdelaziz, Ayush Khurana, Alexander Chase, Diana A. Gorog, Rajiv Das, Azfar Zaman, Mohammad AlKhalil, Mohamed Farag

**Affiliations:** ^1^ Cardiothoracic Department Freeman Hospital Newcastle Upon Tyne UK; ^2^ Keele Cardiovascular Research Group, Centre for Prognosis Research, Institute for Primary Care and Health Sciences Keele University Keele UK; ^3^ Cardiac Department Manchester Royal Infirmary Manchester UK; ^4^ Lancashire Cardiac Centre Blackpool Victoria Hospital Blackpool UK; ^5^ Swansea Bay University Health Board Swansea UK; ^6^ National Heart and Lung Institute Imperial College London UK; ^7^ School of Life and Medical Sciences University of Hertfordshire Hertfordshire UK; ^8^ East and North Hertfordshire NHS Teaching Trust Stevenage UK

**Keywords:** Allegra, aortic stenosis, TAVI, transcatheter aortic valve implantation, valve‐in‐valve

## Abstract

**Introduction:**

The prevalence of bioprosthetic aortic valve failure is increasing due to the broader use of bioprosthetic valves, both surgical and transcatheter, and an ageing population. Registry data indicate a higher risk of coronary obstruction and elevated gradients following valve‐in‐valve transcatheter aortic valve implantation (TAVI). We report outcomes of the Allegra transcatheter heart valve system in valve‐in‐valve TAVI.

**Methods:**

In this retrospective multicentre cohort study, all patients undergoing valve‐in‐valve TAVI with the Allegra system between 2019 and 2025 at five UK centers were included. The combined primary outcomes were technical success, device success, and early safety at 30 days, as per the VARC‐3 (Valve Academic Research Consortium‐3) criteria. A key secondary outcome was all‐cause mortality at the longest documented follow‐up.

**Results:**

A total of 106 patients underwent valve‐in‐valve TAVI. The mean age was 79 ± 6 years, with 57.5% being female. Almost half of the patients had an index valve ≤ 21 mm, with an STS (Society of Thoracic Surgery) score of 7.1% (5.1–12.0). The post‐implant aortic valve mean pressure gradient was 12 ± 6 mmHg, and the mean valve area was 1.7 ± 0.5 cm^2^. The combined primary outcomes were technical success of 86.8% (CI 80.3%–93.2%), device success of 75.5% (CI 67.3%–83.7%), and early safety of 86.8% (CI 80.3%–93.2%). All‐cause mortality at 30 days was 0%. All‐cause mortality occurred in 6.6% at a median follow‐up of 15 months.

**Conclusions:**

The Allegra TAVI system demonstrates promising technical and device success rates, with early safety and good post‐implant haemodynamic performance in high‐risk patients undergoing valve‐in‐valve TAVI. Larger randomized trials are needed to confirm these results.

## 1 Introduction

The prevalence of bioprosthetic aortic valve failure is rising due to increased preference for surgical and transcatheter bioprostheses, coupled with ageing populations and improved post‐implant surveillance [1]. The major advantage of bioprosthetic valves over mechanical valves is the avoidance of lifelong anticoagulation. Nonetheless, a significant limitation of bioprostheses is the higher rate of structural valve deterioration and failure, which may require further reintervention. The rate of surgical bioprosthetic aortic valve failure ranges from 16% at 15 years to over 30% at 20 years [2, 3]. Transcatheter aortic valve failure rates vary, but recent data show low rates of re‐intervention in the short‐ to medium‐term, with around 2.6%–4.3% at 5–10 years [4]. Surgical re‐intervention for bioprosthetic aortic valve failure is associated with 30‐day mortality of up to 10% and 1‐year mortality of 20% in registry data [5]. The availability of transcatheter aortic valve implantation (TAVI) offers a lower‐risk alternative for treating bioprosthetic aortic valve failure, particularly in the elderly and/or comorbid patients [6], and its application has increased over the last decade. Data show that valve‐in‐valve TAVI is associated with lower in‐hospital mortality than redo surgical aortic valve replacement (1.25% vs. 3.4%, respectively) [7].

There is, however, conflicting data on longer‐term outcomes and valve haemodynamic performance following valve‐in‐valve TAVI compared to surgical aortic valve replacement [7]. There are two key considerations when performing valve‐in‐valve TAVI. Firstly, coronary obstruction and secondly, elevated post‐implant gradients [8]. The Global Valve‐in‐Valve registry reported a rate of coronary obstruction between 3% and 4% [9]. The VIVID (Valve‐in‐Valve International Data) registry reported elevated post‐procedural transvalvular gradients in 41.2% of patients with balloon‐expandable valves and 23.4% with self‐expanding valves [10]. One‐year mortality was 15% and 18% with self‐expanding and balloon‐expandable valves, respectively [10].

There are key features of the TAVI valve which contribute to the risk of coronary obstruction. These are neo‐skirt formation, leaflet overhang and sinus sequestration [11]. The creation of a neo‐skirt involves pinning the leaflets of the old valve above the sealing skirt of the new valve, thereby forming a fluid‐impermeable tube graft. This can lead to sinus sequestration if this neo‐skirt extends beyond the sinotubular junction and obstructs the coronary arteries. Leaflet overhang is when the new valve is implanted lower than the old valve, allowing the old valve leaflets to hang above the frame, thereby reducing the height to which the neo‐skirt extends [12].

The Allegra transcatheter aortic valve is composed of a self‐expanding nitinol frame with a sewn‐in bovine pericardial valve in the supra‐annular position [13]. Compared to other contemporary valves, the Allegra valve stent has a concave design and deflects at the tip during diastole to reduce stress on the valve leaflets, potentially prolonging leaflet durability (Figure 1). Initial studies have demonstrated its short‐term safety and haemodynamic performance in native aortic valve implantation [13]. An observational study reported similarly high success rates and a low risk of procedural complications but included only 15 valve‐in‐valve cases [14]. Design improvements have introduced the IMPERIA delivery system, which enables the retrieval and redeployment of the valve. The EMPIRE 1 study showed good results with the new delivery system, including in 16 valve‐in‐valve cases [15]. There are limited studies comparing individuals’ valve types with updated VARC‐3 criteria (Table 1).

**FIGURE 1:**
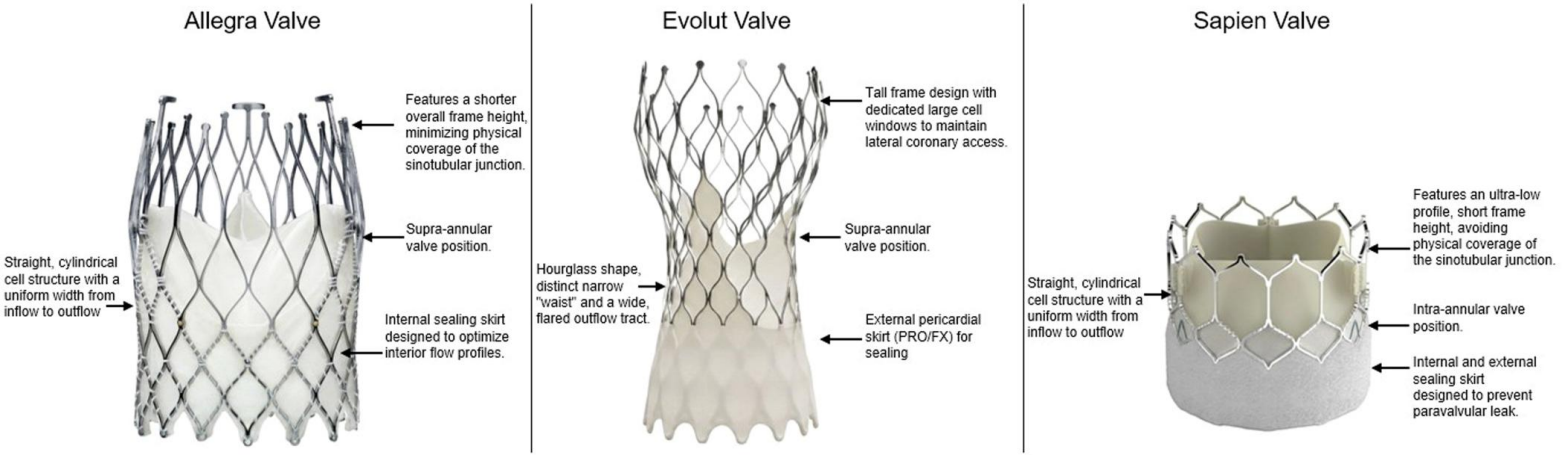
Representation of differences between self‐expanding Allegra and Evolut and balloon‐expandable Sapien valves. [Color figure can be viewed at wileyonlinelibrary.com]

**TABLE 1:** Studies comparing outcomes with Evolut and Sapien valves in patients undergoing valve‐in‐valve TAVI.

Study	Sample size (n)	30‐day mortality	Long‐term mortality	Device success	Technical success	Early safety
Bleiziffer et al. [10, 16]	Evolut 523 Sapien 435	NR	Overall, 16.8% at 1 year and 62% at 8 years	NR	NR	NR
Webb et al. [17]	Sapien 365	2.7%	12.4% at 1 year	NR	NR	NR
Nuche et al. [18]	Sapien 46 Evolut 52	0%	6.5% at 1 year 3.8% at 1 year	NR	NR	NR

We report on the clinical performance and outcome of the Allegra valve in patients with bioprosthetic aortic valve failure undergoing TAVI who were at high surgical risk.

## 2 Methods

### 2.1 Study Design

We undertook a multicentre, retrospective observational study. The study was conducted in accordance with the Declaration of Helsinki and Good Clinical Practice guidelines. Anonymised data were obtained from the institutional database usually utilized for patient care and therefore the need for informed consent was waived by the institutional Medical Ethical Committee. Baseline clinical, echocardiographic, computed tomography (CT) scan, operative and outcome imaging and clinical data were prospectively collected, validated and entered into the database, which was analyzed retrospectively (Table 2).

**TABLE 2:** Baseline patient demographics (*n* = 106).

Age	78.9 ± 6.4
Female	61 (57.5)
BMI (kg/m^2^)	27.9 ± 6.0
Comorbidities	
*Ischemic heart disease*	32 (30.2)
*Previous stroke*	12 (11.3)
*Chronic obstructive pulmonary disease*	19 (17.9)
*Peripheral vascular disease*	10 (9.4)
*Chronic kidney disease*	38 (35.8)
*Permanent pacemaker*	9 (8.5)
Electrocardiogram	
*Sinus rhythm*	68 (64.2)
*Atrial fibrillation*	38 (35.8)
*Left bundle branch block*	11 (10.4)
*Right bundle branch block*	18 (17)
Transthoracic echocardiogram	
*Aortic valve maximum velocity (m/s)*	4.05 ± 0.84
*Aortic valve area (cm* ^2^ *)*	0.87 ± 0.41
*Indexed effective orifice area (cm* ^*2*^ */m* ^*2*^ *)*	0.48 ± 0.23
*Aortic valve mean pressure gradient (mm Hg)*	43.3 ± 15.4
*Aortic valve peak pressure gradient (mm Hg)*	70.7 ± 23.2
*Left ventricular ejection fraction (%)*	51.6 ± 8.6
*Severe transvalvular aortic regurgitation*	33 (31.1)
Computed tomography	
*Left coronary ostium height (mm)*	9.9 ± 3.8
*Left coronary ostium height* < *10 mm*	58 (54.7)
*Left valve‐to‐coronary distance (mm)*	6.1 ± 1.9
*Left valve‐to‐coronary distance* < *4 mm*	10 (9.4)
*Right coronary ostium height (mm)*	12.7 ± 4.5
*Right coronary ostium height* < *10 mm*	28 (26.4)
*Right valve‐to‐coronary distance (mm)*	5.9 ± 2.2
*Right valve‐to‐coronary distance* < *4 mm*	18 (17)
*Left and right coronary ostium height* < *10 mm*	24 (22.6)
*Left and right valve‐to‐coronary distance* < *4 mm*	3 (2.8)
Horizontal aorta (aortic valve angle > 50°)	17 (16.3)
EuroSCORE II (%)	7.0 (4.6–9.2)
STS Score (%)	7.1 (5.1–12.0)

*Note:* Values are mean ± standard deviation, median (IQR) or n (%). Missing data < 10% in each domain.

Abbreviations: BMI: Body Mass Index; STS: Society of Thoracic Surgeons. Missing data < 10% in each domain.

### 2.2 Patient Selection

Patients were retrospectively included from five high‐volume TAVI centers in the UK between November 2019 and November 2025 (97% of cases after 2022). All patients provided written informed consent for the valve‐in‐valve TAVI procedure using the Allegra valve, and after referral by the Heart Team. All patients with previous bioprosthetic aortic valves were included, and patients with metallic valves were excluded.

### 2.3 Study Device and Valve Implantation

The Allegra TAVI System (NVT GmbH, Hechingen, Germany) offers a novel valve design and delivery system. The valve comes in 23, 27, and 31 mm sizes and is delivered via a commercially available 18 Fr sheath. The new IMPERIA delivery system allows easy commissural alignment and enables the recapture, reposition, or retrieval of the Allegra valve, ensuring predictable deployment with no significant haemodynamic compromise. Selection of valve size across centers was standardized using computerized tomography (CT)‐based sizing.

### 2.4 Study Outcomes

The combined primary outcomes were technical success upon exit from the procedure room (defined as a composite of freedom from mortality, successful access, delivery of the device and retrieval of the delivery system, correct positioning of the valve and freedom from surgery or major intervention related to the device or cardiac structural complication or vascular access related complication); device success at 30 days (defined as a composite of technical success, freedom from mortality, freedom from surgery or major intervention related to the device or cardiac structural complication or vascular access related complication and intended performance of the bioprosthetic valve at 30 days); and early safety (defined as a composite of freedom from all‐cause mortality at 30‐days, freedom from stroke, freedom from VARC (Valve Academic Research Consortium) 2–4 bleeding, freedom from major vascular or access‐related or cardiac structural complications, freedom from acute kidney injury stage 3 or 4, freedom from moderate or severe aortic regurgitation (confirmed by transthoracic echocardiogram), freedom from new permanent pacemaker and freedom from surgery or intervention related to the device at 30‐days), as per the VARC‐3 criteria [19]. Secondary outcomes were all‐cause mortality up to 30 days and longest documented follow‐up, as well as the individual components of the aforementioned outcomes, including vascular complications requiring further intervention, significant bleeding requiring blood transfusion, new pacemaker implantation, heart failure hospitalization, stroke confirmed on brain imaging, coronary artery occlusion and valve embolization rates at 30 days. Valve thrombosis rates confirmed on CT scan were also captured during the study follow‐up.

### 2.5 Statistical Analysis

Statistical analyses were performed using SPSS, Version 27 (IBM Corp., Armonk, NY, USA). Continuous variables were expressed as mean ± standard deviation or median (interquartile range). Categorical variables were expressed as frequencies and percentages. Multivariable Cox regression analysis was performed to assess the interaction of all prognostically significant baseline, key procedural and post‐procedural variables on all‐cause mortality at the longest follow‐up. Kaplan‐Meier analysis was used to estimate freedom from mortality at the longest follow‐up.

## 3 Results

### 3.1 Baseline Demographics and Imaging Features

A total of 106 patients who underwent valve‐in‐valve TAVI procedures using the Allegra system were included (Figure 2). The baseline demographics, imaging and index valve data are shown in Table 2. The mean age was 79 ± 6 years, and 57.5% were female. The mean EuroSCORE II and STS (Society of Thoracic Surgery) scores were 7.0% (4.6–9.2) and 7.1% (5.1–12.0), respectively. Magna Ease (23.6%), Trifecta (20.7%) and Perimount (19.8%) accounted for 64% of failed index bioprostheses, and 48.1% of patients had a valve ≤ 21 mm, with a mean valve size of 22 ± 2 mm (Table 3).

### 3.2 Procedural Characteristics

The earlier delivery system (Permaflow) was used in 93 cases, and the current IMPERIA delivery system was used in 13 cases. Successful device implantation occurred in 100 (94.1%) cases. There were six cases where a second valve was required (Table 4). The Allegra valve demonstrated good haemodynamic performance post‐implant (Figure 3). No patients had transvalvular leak, and one patient had a residual moderate‐to‐severe paravalvular leak.

**TABLE 3:** Index aortic valve prosthesis type and size (*n* = 106).

	Number (%)
**Type**	
Magna Ease	25 (23.6)
Trifecta	22 (20.7)
Perimount	21 (19.8)
Crown	8 (7.5)
Mitroflow	8 (7.5)
Hancock II	6 (5.7)
Edwards Sapien 3	6 (5.7)
Perceval	4 (3.8)
Mosaic	2 (1.9)
Epic	2 (1.9)
Direct Flow Medical	1 (0.9)
Biovalsalva Conduit	1 (0.9)
**Size**	
19 mm	9 (8.5)
20 mm	1 (0.9)
21 mm	41 (38.7)
23 mm	36 (33.9)
25 mm	9 (8.5)
26 mm	2 (1.9)
27 mm	3 (2.8)
29 mm	1 (0.9)
S	1 (0.9)
M	2 (1.9)
XL	1 (0.9)

*Note:* Values are *n* (%).

**FIGURE 2:**
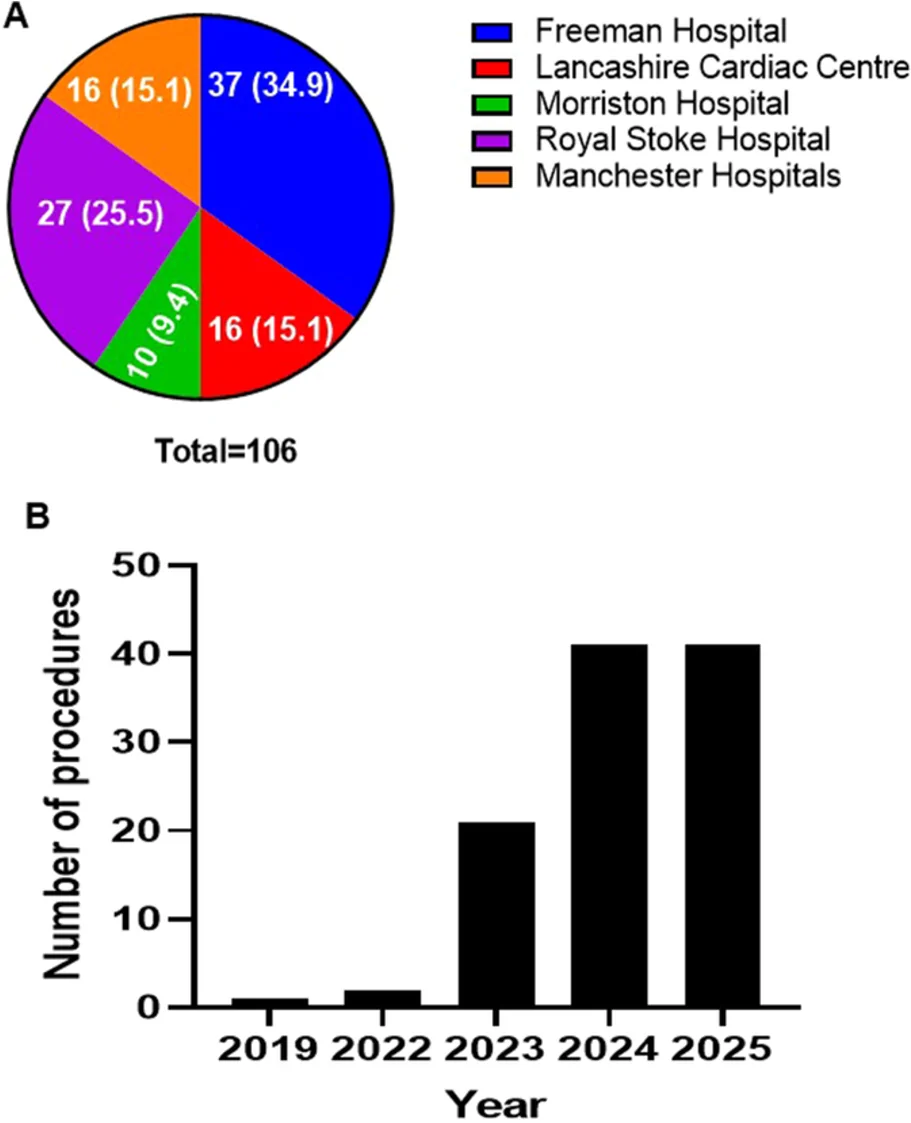
Number of procedures performed. (A) Depicts the number of procedures performed by center. (B) Depicts the procedure volume by year. [Color figure can be viewed at wileyonlinelibrary.com]

### 3.3 Clinical Outcomes

The combined primary outcomes of technical success occurred in 92 (86.8%, CI 80.3–93.2) patients, device success in 80 (75.5%, CI 67.3–83.7) patients, and early safety in 92 (86.8%, CI 80.3–93.2) patients. Device failure was noted in six cases requiring a second valve, four cases of coronary obstruction successfully treated by chimney stenting, three cases of vascular complications requiring surgical intervention (two of which required blood transfusion), one case of left ventricular perforation (secondary to support wire) requiring pericardiocentesis and blood transfusion, one case of residual moderate‐severe paravalvular aortic regurgitation post‐implant and 12 cases of elevated transvalvular aortic valve gradients on post‐implant transthoracic echocardiography (mean pressure gradient of ≥ 20 mmHg).

The secondary outcome of all‐cause mortality occurred in 6.6% of cases at a median follow‐up of 15 (8–22) months (Table 5 and Figure 4). No patients died within 30 days, and there was no conversion to cardiac surgery. Valve thrombosis occurred in two patients (1.9%) during the follow‐up period and resolved with anticoagulation therapy. Cox regression analysis revealed no significant interactions between the assessed variables and all‐cause mortality at the longest follow‐up (Table 6). There was no difference in all‐cause mortality based on index AVR ≤ 21 mm compared to > 21 mm (*p* = 0.068).

**TABLE 4:** Procedural characteristics (*n* = 106).

Access Route	
Femoral	103 (97.2)
Subclavian	3 (2.8)
Predilatation	79 (74.5)
Predilatation balloon size (mm)	17.2 ± 5.4
Post‐dilatation	56 (52.8)
Post‐dilatation balloon size (mm)	16.9 ± 8.6
Valve Fracture	6 (5.7)
Chimney Stenting	4 (3.8)
Invasive peak‐to‐peak pressure gradient pre‐implant *	51.7 ± 29.7
Invasive peak‐to‐peak pressure gradient post‐implant *	6.1 ± 6.5
Contrast volume (ml)	76 ± 49
Procedure length (minutes)	88 ± 54
Imperia Delivery System	13 (12.3)
Allegra valve size	
23 mm	92 (86.8)
27 mm	10 (9.4)
31 mm	4 (3.8)
Second valve implanted	6 (5.7)
Unable to align inside index valve > changed to Evolut and Navitor valves	2 (1.9)
Unable to cross small Perceval valve > changed to Evolut valve	1 (0.9)
Unable to cross severely calcified/angulated aortic arch > changed to SAPIEN valve	1 (0.9)
Embolized 31 mm valve from rotated index valve > changed to SAPIEN valve	1 (0.9)
Broken Permaflow delivery system > changed to Navitor valve	1 (0.9)
Post‐implant transthoracic echocardiogram	
*Aortic valve maximum velocity (m/s)*	2.33 ± 0.54
*Aortic valve area (cm* ^*2*^ *)*	1.68 ± 0.46
*Aortic valve mean pressure gradient (mm Hg)*	12.3 ± 5.9
*Aortic valve peak pressure gradient (mm Hg)*	22.4 ± 9.9
*Left ventricular ejection fraction (%)*	51.9 ± 9.5
Moderate‐severe paravalvular leak	1 (0.9)
Days before discharge	4.9 ± 9.2
Median follow‐up (months)	15.3 (8.1–21.6)

*Note:* Values are mean ± standard deviation, Median (IQR), or *n* (%). Missing data < 10% in each domain, apart from * invasive gradients 25%.

**FIGURE 3:**
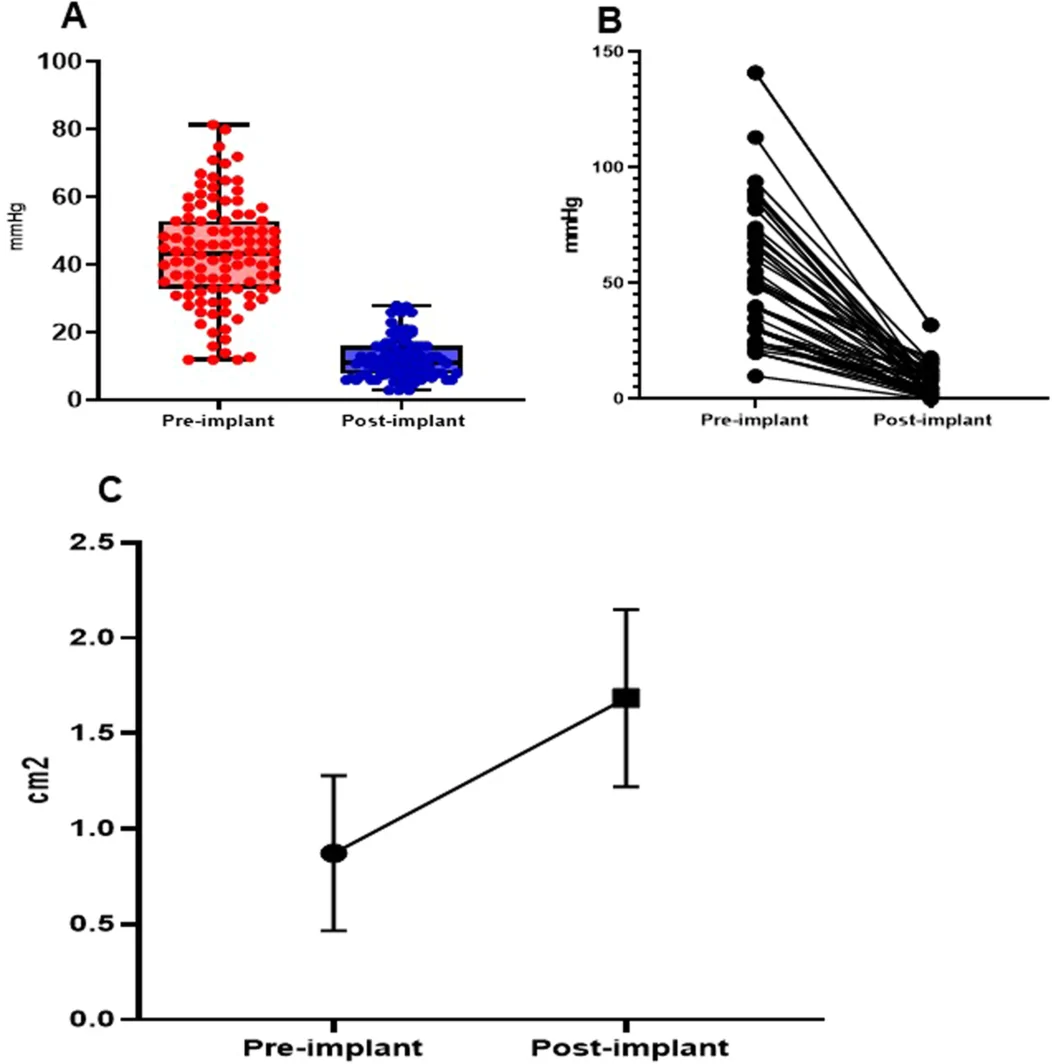
Allegra valve haemodynamics. (A) Mean aortic transvalvular pressure gradients pre‐ and post‐implant on echocardiogram. (B) Invasive peak‐to‐peak pressure gradients pre‐ and post‐implant. (C) Change in mean aortic valve area pre‐ and post‐implant on echocardiogram. [Color figure can be viewed at wileyonlinelibrary.com]

**TABLE 5:** Clinical outcomes of the Allegra system (*n* = 106).

**Co‐primary outcomes at 30 days**	
Technical success	92 (86.8)
Device success	80 (75.5)
Early safety	92 (86.8)
**Reasons for technical failure**	
Second valve implanted due to failure to cross valve, failure to align the valve, embolization of valve, or broken delivery system	6 (5.7)
PCI due to coronary obstruction	4 (3.8)
Left ventricular perforation and tamponade due to support wire	1 (0.9)
Vascular complications requiring further intervention (VARC‐3 criteria)	3 (2.8)
**Clinical outcomes at 30‐day**	
All‐cause mortality	0 (0)
Vascular complications requiring further intervention (VARC‐3 criteria)	3 (2.8)
Major bleeding requiring blood transfusion	3 (2.8)
New pacemaker implantation	3 (2.8)
Heart failure hospitalization	3 (2.8)
Stroke confirmed on brain imaging	1 (0.9)
Coronary artery obstruction	4 (3.8)
Valve embolization	1 (0.9)
**Clinical outcomes at a median follow‐up of 15 months**	
All‐cause mortality	7 (6.6)
Valve thrombosis	2 (1.9)

*Note:* Values are *n* (%).

Abbreviations: PCI, percutaneous coronary intervention; VARC‐3, Valve Academic Research Consortium 3.

**TABLE 6:** Multivariable Cox regression analysis of all‐cause mortality.

	Hazard Ratio	95% Confidence Interval
Age	0.971	0.842 to 1.120
Female	1.524	0.271 to 8.569
Body Mass Index	1.016	0.976 to 1.057
Previous Pacemaker	1.807	0.853 to 3.827
Previous Stroke	0.489	0.028 to 8.614
Chronic Obstructive Pulmonary Disease	1.233	0.139 to 10.953
Peripheral Vascular Disease	1.023	0.435 to 2.610
Ischemic Heart Disease	0.485	0.083 to 2.817
Chronic Kidney Disease	1.265	0.250 to 6.399
Structural Valve Deterioration (VARC‐3)	0.915	0.468 to 1.789
Imperia vs Permaflow systems	1.246	0.786 to 1.898
Index AVR ≤ 21 mm	0.133	0.015 to 1.156

*Note:* Values are reported as hazard ratios and 95% confidence intervals.

Abbreviations: AVR, Aortic Valve Replacement; VARC‐3, Valve Academic Research Consortium‐3.

**FIGURE 4:**
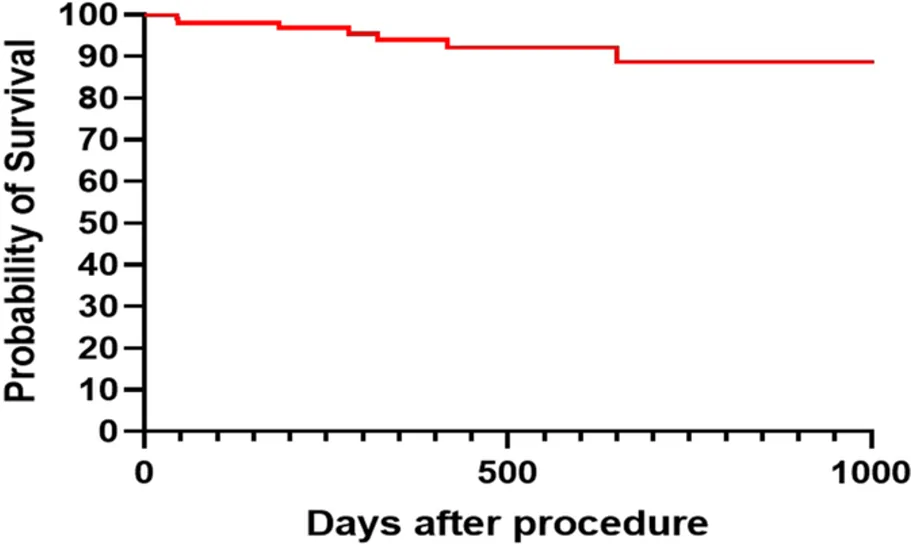
Kaplan‐Meier graph of all‐cause mortality after Allegra valve implantation. [Color figure can be viewed at wileyonlinelibrary.com]

## 4 Discussion

This is the largest registry to date of valve‐in‐valve TAVI cases treated with the Allegra TAVI system and demonstrating good clinical and haemodynamic outcomes. The study population had a high surgical risk, broadly comparable to that of the cohort studied in the original PARTNER trial [20]. They were predominantly female, likely attributable to the recognized higher prevalence of prosthesis‐patient mismatch in female patients [21]. Almost half of the patients had an index valve size of ≤ 21 mm, reflecting the smaller anatomy in this cohort. The majority of cases had elevated trans‐aortic valve gradients, with significant concomitant aortic regurgitation in approximately a third. The height of the coronary ostia and their distance from the index valve were compromised in a substantial proportion of cases (Table 2), further complicating the valve‐in‐valve TAVI procedure.

### 4.1 Device Success and Clinical Outcomes

The Allegra TAVI system demonstrated technical success rates of 86.8%, device success rates of 75.5%, and early safety rates of 86.8% according to the VARC‐3 criteria in this high‐risk cohort. Overall complication rates in our cohort were low (Table 5). There were no deaths either in‐hospital or up to 30 days. All‐cause mortality at a median follow‐up of 15 months was 6.6%. A recent registry of valve‐in‐valve TAVI, predominantly using the CoreValve and Sapien systems, reported an overall technical success of 80.3%, a device success of 55.9%, and an early safety of 67.4% according to VARC‐3 criteria [22]. Another registry data of valve‐in‐valve TAVI reported a 1‐year mortality of 14.7% in a similar group of high‐risk patients [23]. The largest VIVID registry reported 30‐day mortality of 7.6%, 1‐year mortality of 16.8%, major vascular complications of 9.2%, pacemaker implantation of 8.3%, and coronary obstruction in 2% of patients [10]. The novel design of the Allegra TAVI system, which allows unobstructed implantation, might have contributed to the absence of in‐hospital/early mortality by preventing haemodynamic obstruction during implantation [24].

### 4.2 Haemodynamic Performance

The haemodynamic profile of the Allegra valve demonstrated good performance in patients with bioprosthetic aortic valve failure. The post‐implant mean aortic valve pressure gradient was 12 ± 6 mmHg, and the mean aortic valve area was 1.7 ± 0.5 cm [2]. Post‐implant moderate‐to‐severe paravalvular regurgitation was observed in one case, likely present pre‐TAVI and misinterpreted as transvalvular. The remaining cohort had either no residual paravalvular/transvalvular aortic regurgitation or only mild on follow‐up echocardiography. Elevated transvalvular gradients, as defined by the VARC‐3 criteria, were present in 11.3% of patients compared to roughly 30% in a recent registry [22], and 26.8% in the VIVID registry [10]. The latter demonstrated that in patients with pre‐existing severe prosthesis‐patient mismatch, the rate of significantly elevated transvalvular gradients was 47.5% compared with 29.6% in those with moderate or less prosthesis‐patient mismatch [25]. The PARTNER 2 valve‐in‐valve registry reported a post‐procedure mean aortic valve pressure gradient of 17 mmHg and a mean aortic valve area of 1.16 cm^2^, as well as severe prosthesis‐patient mismatch in 58.4% of cases [17]. This registry data shows that the Allegra valve offers favorable post‐procedural gradients in the most challenging anatomies of patients undergoing valve‐in‐valve TAVI.

In summary, this real‐world registry of patients undergoing valve‐in‐valve TAVI for bioprosthetic valve failure shows the safety and efficacy of the Allegra valve. As TAVI becomes more prevalent in younger patients, transcatheter valve design will need to adapt to safe use in valve‐in‐valve implantation. This registry also confirms a higher preponderance of bioprostheses failure in female subjects and highlights the need for targeted research in this vulnerable population.

## 5 Limitations

This multicentre retrospective cohort study is limited by its small sample size and observational design. Also, although data were analyzed from a prospective registry of consecutive real‐world patients undergoing valve‐in‐valve TAVI using the Allegra system, all inherent biases of a retrospectively evaluated, unmonitored, multicentre registry should be considered. Moreover, the lack of core‐lab adjudication of echocardiographic data, as well as heterogeneity in imaging protocols, might have increased interobserver variability in reported findings. Nonetheless, all included centers broadly use and report echocardiograms in accordance with the British Society of Echocardiography (BSE) guidelines [26]. Furthermore, the 15‐month follow‐up period is too short to meaningfully assess longer‐term structural valve deterioration beyond 2 years. Additionally, the Allegra delivery system was changed from the previous Permaflow to the improved IMPERIA, which might have affected earlier valve delivery and implantation.

## 6 Conclusions

The Allegra TAVI system demonstrates promising technical and device success rates, with early safety and favorable post‐implant haemodynamic performance in high‐risk patients with bioprosthetic aortic valve failure. Larger randomized trials compared with other valve platforms are needed to validate these results.

## Funding

The authors have nothing to report.

## Conflicts of Interest

K.R. received proctor and speaker fees from Biosensors. A.K. received proctor fees and honorarium from Abbott, Meril and Medtronic. A.C. received proctor fees from Biosensors and Abbott. M.A. received proctor fees from Biosensors. M.F. received proctor fees from Abbott and Meril. M.F. received speaker fees and honoraria from Edwards, Medtronic, and Biosensors. No other authors declare conflicts of interest.

## Data Availability

The data that support the findings of this study are available from the corresponding author upon reasonable request.
